# Dual Challenge of Cancer and COVID-19: Impact on Health Care and Socioeconomic Systems in Asia Pacific

**DOI:** 10.1200/GO.20.00227

**Published:** 2020-06-26

**Authors:** Roselle De Guzman, Monica Malik

**Affiliations:** ^1^ Oncology Unit, Manila Central University–Filemon D. Tanchoco Medical Foundation Hospital, Caloocan City, the Philippines; ^2^ Department of Radiation Oncology, Nizam’s Institute of Medical Sciences, Hyderabad, India

## Abstract

Coronavirus or COVID-19 is caused by severe acute respiratory syndrome coronavirus 2. The COVID-19 pandemic has resulted in social and economic disruption throughout the entire world. Each country is being challenged. Although much of the world’s focus has been on the rapid spread in Italy, Spain, and the United States, the potential impact on the world’s poor, a majority of whom are living in Asia, could be devastating. Asia has the world’s most densely populated cities, and its developing countries are facing challenges in their socioeconomic and health care systems. COVID-19 is quickly overwhelming the fragile and overstretched health systems of low- and low- to middle-income countries. With its aging population having chronic diseases and the growing burden of cancer, Asia is facing the dual challenge of controlling the spread of COVID-19 and at the same time providing and maintaining cancer care.

## INTRODUCTION

The Asia Pacific region has experienced extreme disasters and events that have had great impacts on the economy, lives, and livelihoods. In 1997, Asia was in a financial crisis. In 2003, the Severe Acute Respiratory Syndrome (SARS) outbreak occurred. Asia has frequently been hit by natural disasters, including the Indian Ocean earthquake in 2004, the Nepal earthquake in 2015, Cyclone Gita in 2018, and the eruption of the Taal volcano in 2020.

**CONTEXT**

**Key Objective**
How does the diverse Asia Pacific region take up the challenge of the COVID-19 pandemic while bearing its growing burden of cancer?
**Knowledge Generated**
The developed and developing countries of the region are responding distinctly and implementing control measures and innovative strategies differently based on their resources and capacities. The vulnerabilities and inequalities that exist in the population of low- and low- to middle-income countries demonstrate how the dual challenge is further stretching health care services and deeply disrupting socioeconomic systems.
**Relevance**
To assist Asia Pacific countries to cope with the short- and long-term impacts of the COVID-19 pandemic, international and regional cooperation and partnerships should continue.

On December 31, 2019, the first cases of COVID-19 detected in Wuhan City, Hubei Province, China, were reported to the WHO.^1^ As early as January 2020, there had been reported cases in Thailand and the United States. The outbreak quickly evolved into a global pandemic in March. COVID-19 cases started rising in Iran, Italy, and the Republic of Korea in February and March. The disease has spread around the world at an alarming speed and has overwhelmed even the most resilient health and social care systems.

The current pandemic is the worst in a century. It has had a major impact on the global economy. Asia Pacific is bearing the brunt of the impact on life, the economy, and society. The human costs are significant. The number of cases is increasing every day and changing every minute. This trend has made the whole world enter lockdown mode. It is certain that incidence and mortality figures will have changed when this publication goes online.^2^

## ECONOMIC AND SOCIAL IMPACTS OF COVID-19 PANDEMIC IN ASIA PACIFIC

The world is facing extraordinarily challenging times. The travel restrictions, community quarantines, and lockdowns have had extensive social and economic impacts in Asia Pacific. The region has more than two thirds of the global population, as well as massive industrial activity, energy supply, and energy consumption. The crisis has shown how the Asia Pacific region is integrated into the world socioeconomic system. It has demonstrated that the brisk action and firm policies of some countries in Asia have addressed COVID-19 effectively and efficiently. In contrast, it has also revealed that poorer countries with developing economies and weak health systems are faced with bigger challenges.

Travel bans are affecting trade and tourism, both domestically and internationally. This is significant for the economies of countries where a significant amount of revenue is generated by tourism. According to the Asian Development Bank (ADB), international tourism receipts provide > 40% of the gross domestic product (GDP) in the Maldives and Palau, for example, and total travel and tourism receipts, including domestic tourism, exceed 10% of the GDP in almost half of the developing member countries (DMCs) of the ADB.^3^ Cambodia, Fiji, and Thailand are the ADB DMCs greatly affected by the COVID-19 pandemic. These countries all have significant tourism industries.^3^

Asia is the world’s manufacturing hub. People from rural areas migrate to big cities to work in factories and other manufacturing industries. As reported by the ADB, 80,000 people in Bangkok who feared lockdown or were out of work had crammed into a single bus station to travel back to their home provinces in March 2020. In India, tens of thousands of migrant workers rushed in panic to return to their villages, fearing unemployment or starvation during the lockdown, because all nonessential work in factories and industries had been halted. Garment factory workers in Bangladesh, Cambodia, Indonesia, and Myanmar are mostly living in crowded housing. Many factories have shut down because of order cancellations from major brands.^3^

Growth in Asia is expected to stall at 0% this year, according to the Asian Development Outlook. This is considered to be the worst growth performance in almost 6 decades. The growth performance during the global financial crisis was 4.7%, and during the Asian financial crisis, it was 1.3%.^4^ The ADB estimates that the global economic losses will be between 2 trillion and 4.1 trillion US dollars. The ADB has provided a $20 billion package that will support and empower Asia Pacific governments and businesses to address the severe economic fallout, respond to the urgent needs of the vulnerable, sick, and poor, and provide vital support for a secure and stable recovery.^4^

## HEALTH CARE SYSTEMS OF LOW- AND LOW- TO MIDDLE-INCOME COUNTRIES IN ASIA PACIFIC: HEALTH INEQUALITIES AND CHALLENGES OF ADOPTING CORONAVIRUS PRECAUTIONS

Although most of the response and media coverage have been focused on China and high-income countries, controlling and preventing the spread of coronavirus in low- and low- to middle-income countries (LMICs) is just as important. The critical circumstances of LMIC health care systems support the fact that their COVID-19 response cannot take the same path as their advanced-economy counterparts.

With no vaccination available, nonpharmaceutical interventions are recommended as feasible ways to suppress the spread of COVID-19 and lower the death rate. Social distancing, hand washing, and shielding of vulnerable groups, including those with preexisting health conditions, the elderly, and pregnant women, are particularly important in the context of fragile health systems. The success of these nonpharmaceutical safety measures against coronavirus relies on widespread compliance, which is additionally challenging to achieve in LMICs. Many of these challenges are driven by economic insecurity and poverty.

Health inequalities are evident in Asia Pacific. As reported by the United Nations Economic and Social Commission for Asia and the Pacific, there are 400 million people living below the international poverty line of US$1.90 per day. There are more than 1 billion who live on less than $3.20 a day.^6^ They often live in urban slums or crowded flats. For many households in low-income countries, the substantially higher rates of overcrowding put people at greater risk of household transmission. Many work in crammed markets or factories where hygiene is not always a priority. Those who are age ≥ 70 years live with an average of 4 individuals age < 70 years. These younger family members are mostly mobile and not always able to limit social distancing; thus, it becomes more difficult to shield the older family members. In addition, many people in low-income countries do not always have access to hand washing facilities or soap.

Most countries in Asia Pacific have higher out-of-pocket health care expenditures. Bhutan, China, Georgia, the Maldives, Sri Lanka, and Thailand are some of the few developing countries with established universal health care systems. The fragile, overburdened health care systems in most LMICs are particularly prone to severe disruption by natural and manmade disasters.^7^ Emergency preparedness for cancer care is usually nonexistent in most LMICs.

LMICs have high rates of infectious diseases like malaria and tuberculosis, as well as higher rates of noncommunicable diseases such as cardiovascular diseases and cancer. Failure to control the spread of coronavirus would potentially place millions of lives at risk.

## ASIA’S AGING POPULATION: BEARING THE BRUNT OF COVID-19

Asia Pacific is rapidly aging. Fifty-eight percent of the global population (ie, 614 million) age > 60 years live in the region. A study from the National Health Commission in China reported that approximately 80% of people who have died as a result of the virus in the country were age > 60 years, and 75% had preexisting conditions.^8^ There are additional risk factors for severe COVID-19 disease that are unique to low-income countries. These are HIV, tuberculosis, chronic obstructive pulmonary disease, rheumatic heart disease, and cardiomyopathies.^9^ Mortality increases with age, and those age > 80 years have the highest mortality.

Participation in the informal labor force is a necessity for many older people, with approximately 40% of those age > 60 years working in countries such as Bangladesh, the Philippines, and Vietnam. Social protection programs must recognize this and provide initiatives to protect against economic disruption.

An age perspective should be included in the development of national and global planning for COVID-19. The design of COVID-19 response programs must identify and recognize the situations and diversity of the older population.

## COPING WITH DUAL CHALLENGES: CANCER AND COVID-19

More than 2 million people are diagnosed with cancer annually in Southeast Asia.^10^ Patients with cancer are more susceptible to infection because of the immunosuppressive state caused by both anticancer treatment and surgery.^11-13^ This vulnerable population is faced with the double-edged sword of the increased risk of potentially more severe COVID-19 infection versus the consequences of delaying effective anticancer therapies. A recent study from He et al^14^ shows that the risk of developing severe events in COVID-19 disease is significantly higher in patients with cancer. A meta-analysis of 11 studies found that the overall pooled prevalence of cancer in patients with COVID-19 was 2.0% (95% CI, 2.0% to 3.0%; *I*^2^ = 83.2%).^15^

In the midst of the COVID-19 pandemic, oncologists weigh the risks of morbidity and mortality resulting from COVID-19 against the magnitude of benefit of intended cancer therapies. Early estimates from China suggest an overall case fatality rate (CFR) of 2%, increasing to 8% for patients age 70 to 79 years and 15% for those age ≥ 80 years.^16^ A report from New York that analyzed 218 patients with cancer diagnosed with COVID-19 showed that the CFR was 37% for hematologic malignancies, 25% for solid cancers, and 55% for lung cancer, suggesting a much higher incidence of severe and fatal infections in patients with COVID-19 with cancer compared with age-adjusted patients without cancer.^17^ A systematic multicenter analysis from 14 hospitals in China showed that patients with lung cancer, metastatic cancer, or hematologic malignancies had the highest frequency of severe events.^18^

There are real challenges in maintaining cancer care while dealing with the demands placed on the health system by COVID-19. LMICs, with their strained resources, have significant preexisting challenges in availability, access, and affordability of cancer care services. Most health care systems face severe disruption of routine services as they struggle to deal with the increasing influx of patients with COVID-19. With strictly enforced confinement measures and suspension of all public transportation facilities, access to tertiary health care can be prohibitive for poor and vulnerable groups of patients. The situation can be worsened by loss of income, unemployment, and lack of access to support from family, caregivers, and friends as mandatory lockdowns take effect.

For patients with cancer, there are significant negative implications when medical attention is diverted exclusively to the COVID-19 situation, overshadowing everyday clinical practice. These include delays in diagnostic procedures and treatment. Reallocation of a number of physicians and nurses to COVID-19 triage and medical care may stretch an already fragile health system. Indeed, many cancer centers faced significant disruption of activities after the outbreak.^19^ The consequences could be far reaching in terms of increased morbidity and mortality.^20^

There can be significant levels of anxiety and stress among patients and health care workers. Ensuring the safety of patients and providers necessitates reducing the number of in-person visits and restriction of visitors from accompanying patients. Patient counseling and palliative care can be immensely challenging, including issues such as how to clarify advance directives and end-of-life care preferences. The demonstration of empathy essential in oncology practice has been made difficult by physical barriers, including facemasks and telemedicine.

The pandemic has also severely disrupted cancer screening, research, and clinical trials.^21^ Multinational clinical trials in oncology can be significantly disrupted as health care systems adapt and various confinement laws are enacted in different countries.^22^

There is a need to continue providing essential health care to the vulnerable while ensuring the safety of both patients and staff during the pandemic. This calls for unprecedented changes in workflow and care delivery models.

## INNOVATIVE WAYS TO MITIGATE NEGATIVE CONSEQUENCES FOR PATIENTS WITH CANCER

The probable survival benefits of receiving cancer treatment still far outweigh the risks of death resulting from COVID-19. The risk of transmitting the new coronavirus is mitigated through effective policies on hospital infection control. These policies ensure the safety of the staff and patients through team segregation and resource allocation, allowing continued care for patients with cancer requiring time-sensitive treatment while avoiding major disruptions to therapeutic clinical studies and education. Triage, prioritization, and modification of workflow treatment protocols when appropriate can be helpful in navigating the crisis. Meticulous screening of patients and staff before permitting entry into outpatient clinics and reorganization of patient flow to minimize contact between patients are important strategies. Waiting times are kept as short as reasonably possible, with careful consideration for patient- and system-level risks of COVID-19.

Hypofractionated irradiation and conversion of intravenous to oral systemic regimens are considered to reduce the number of clinic visits and the associated risks. Postponing adjuvant chemotherapy or elective surgery for less aggressive cancers is also considered. Postponing routine post-treatment monitoring visits, delaying or withholding treatments with uncertain benefits, and avoiding severely toxic or immunosuppressive therapies when appropriate can help mitigate some of the challenges.

Hanna et al^23^ proposed a conceptual framework on how to prioritize cancer treatment during the pandemic. Prioritizing the delivery of therapy will be influenced by the magnitude of potential treatment benefit and therapeutic intent. Other considerations include patient-specific factors, effects of interruptions or delays on outcomes, and availability of staff capable of safely delivering treatments.

For health systems without confirmed COVID-19 cases, continuing most treatments and considering postponement of those without anticipated adverse effects on outcomes are reasonable strategies.

During the pandemic, triage decisions require even more interspecialist coordination and communication than usual. Oncologists report unprecedented levels of cooperation and collegiality across borders. Clinicians in Taiwan and Singapore are sharing their experiences with those in the Philippines and other developing countries in Asia. The oncology community shares strategies and reports deriving comfort and professional solidarity from these interactions.

## ASIA’S CAPACITY, RISKS, AND HUMANITARIAN SETTINGS

Asia encompasses all levels of economic development and infrastructure. Japan, Singapore, South Korea, and Taiwan have fully developed economies. Malaysia and Thailand have good infrastructure in urban areas but less developed infrastructure in rural areas. Bangladesh, China, India, Indonesia, and Pakistan, the most heavily populated countries in Asia, have weak public health infrastructure. The initial main hurdle in this pandemic is the lack of testing for COVID-19. There has been inadequate COVID-19 testing capacity in some countries in Asia.

More testing prevents new infections and saves lives. Commended globally as success stories in COVID-19 response, Taiwan and Singapore have engaged in aggressive testing, contact tracing, and containment approaches, which have been highly effective. Unfortunately, with a GDP per capita between US$300 and US$4,000, most LMICs lack sufficient testing and monitoring resources to effectively replicate a test-track-contain–based response.^5^ COVID-19 cases may either be undetected or underdetected in developing countries with fewer resources. India, Indonesia, and Pakistan initially had few confirmed cases despite having large populations.

Deep Knowledge Group, a consortium of commercial and nonprofit organizations, has developed advanced frameworks to efficiently analyze data from the WHO, Johns Hopkins University, the US Centers for Disease Control and Prevention, Worldometers, and other publicly available sources.^24^ The risk and safety rankings are based on infection spread risk, government management, health care efficiency, regionally specific risks, quarantine efficiency, monitoring and detection, and emergency treatment readiness. The results provide insights necessary to help people and governments make informed decisions to maximize beneficial outcomes for humanity.

According to the data analyzed by Deep Knowledge Group, Asia Pacific countries with high levels of safety are South Korea, Australia, China, New Zealand, Taiwan, Singapore, Japan, and the Chinese special administrative region of Hong Kong.^24^ Those with low safety levels are Myanmar, Cambodia, Sri Lanka, Nepal, Laos, Bangladesh, Indonesia, and the Philippines.^24^

## ACCELERATING GLOBAL RESEARCH AND RESEARCH SUPPORT IN THE REGION

Since the outbreak of SARS, the H1N1 influenza pandemic, and the Ebola outbreak, authoritative guidance on the conduct of research has been produced. This guidance on how to conduct ethical research during emergencies should be adhered to by researchers, funders, review bodies, manufacturers, and publishers.

From February 11 to 12, 2020, the WHO, in collaboration with the Global Research Collaboration for Infectious Disease Preparedness and Response, organized a global forum on research and innovation for COVID-19. The aim was to coordinate and accelerate global research work to target diseases, develop diagnostics, medicines, and vaccines fast, and promptly respond to outbreaks.

Recognizing the critical importance of the rapid availability of effective vaccines against COVID-19, the WHO-led Solidarity trial was announced on March 18, 2020.^25^ This trial is an unprecedented global effort to compare the safety and effectiveness of 4 treatment protocols using different combinations of remdesivir, lopinavir/ritonavir, interferon beta, chloroquine, and hydroxychloroquine. As of April 21, 2020, > 100 countries were participating, including Asia Pacific countries Brunei Darussalam, Indonesia, Malaysia, Myanmar, North Korea, the Philippines, South Korea, and Thailand.

The COVID-19 Clinical Research Coalition was formed to conduct research on the prevention and treatment of COVID-19 in resource-poor settings.^26^ The coalition brings together an unprecedented array of health experts, public sector research institutes, academia, ministries of health, international organizations, nongovernmental organizations, and funders, all committed to finding COVID-19 solutions for LMICs. The coalition takes advantage of existing multinational and multidisciplinary expertise in running clinical trials in resource-poor settings.

## DIGITAL INFRASTRUCTURE

The spread of COVID-19 to every continent within weeks is a result of the digital and technologic revolution that has transformed the world. As health care systems are being challenged by the growing number of COVID-19 cases, health care delivery is being transformed and systems scaled up by digital technologies.

Although these systems have been in existence for a while, the pandemic has pushed them into the mainstream of health care delivery, with the aims of reducing risks to patients and health care workers and mitigating barriers to care access. In India, there were no laws on the practice of telemedicine until March 2020, when the Indian Ministry of Health drafted guidelines to allow providers to harness the full potential of technology in health care delivery. In telemedicine, various aids can be used, such as video and audio software and apps, online chat and messaging platforms, and e-mail.

As health care systems transition to digital modes of communication, issues such as lack of access to the Internet and smartphones, lack of time and training for effective teleconsultation, lack of physical examination, and issues with patient consent can result in inadequate sharing of information and reduced satisfaction. Studies have shown that the quality of these consults can be improved with training and use of predesigned formats.^27^

### How Digital Contact Tracing Slowed COVID-19 in East Asia

China, Singapore, South Korea, Taiwan, and Japan are flattening the curve and slowing the spread of COVID-19. They are actively using technology to gather data on the progress of the virus, containment efforts, and tracking of the infection status, movements, and contacts of infected individuals.

Singaporeans are encouraged by the government to install TraceTogether. This is a faster contact tracing method that exchanges Bluetooth signals between cellular phones in close proximity. The Hong Kong government also has an effective containment strategy, requiring each new overseas arrival to download the StayHomeSafe app. A paired wristband uses geofencing technology that detects and analyses environmental communication signals and geospatial signals, identifying those who violate quarantine. South Korea has developed apps that supplement official government contact tracing efforts. The Corona 100m app collects data from public government sources, alerting users of any diagnosed patient with COVID-19 within a 100-meter radius. Information includes the patient’s diagnosis date, age, sex, nationality, and prior locations. Taiwan has also been lauded for its COVID-19 containment, using mobile phone tracking to enforce quarantines.

### Leveraging Telemedicine for Cancer Care

Amid the ongoing pandemic, telemedicine is offering a preventive strategy to keep vulnerable patients with cancer safe.^28^ Moving cancer care to telehealth is a way of reaching out to and connecting with patients, especially with governments imposing strict lockdowns and suspending public transportation. Technology does not replace an in-person visit, but it presents a beneficial substitute in cases where that is not feasible.^29^

Telemedicine can also support the endeavor to mitigate patient anxiety and alleviate fears. It can significantly reduce health care costs and demand for personal protective equipment. Virtual multidisciplinary case conferences remain important venues to prioritize the care of patients and review policies and adopt guidelines. Feedback from stakeholders suggests increasing satisfaction with the transition to virtual tumor boards, which can also work toward supporting hub-and-spoke models of care and facilitate multidisciplinary coordination.^30^

Concerns about online security and privacy mandate effective measures by all stakeholders, including technology companies, to step up efforts in this direction. Many governments and professional societies now recommend that digital modes of health care delivery be used whenever feasible and are devising reimbursement models to compensate health care workers for the use of telemedicine for patient care while supporting efforts to train health care workers in the appropriate and efficient use of technology.^31,32^

The power of technology is also being used to offer ongoing education to trainees and staff who may be away because of various reasons like team segregation or reallocation of responsibilities.^33^

## REGIONAL COOPERATION TO BUILD RESILIENT AND SUSTAINABLE FUTURE

The unprecedented public health emergency caused by the COVID-19 pandemic is taking a heavy toll on Asia Pacific. The region stands at a pivotal moment.

The South Asian Association for Regional Cooperation (SAARC) is the regional intergovernmental organization and geopolitical union of states in South Asia. Its member states are Afghanistan, Bangladesh, Bhutan, India, the Maldives, Nepal, Pakistan, and Sri Lanka.^34^ SAARC established a coronavirus emergency fund to respond to the global coronavirus pandemic and mitigate the risks associated with the pandemic in South Asia.^35^

During a special summit held in April 2020, Association of Southeast Asian Nations (ASEAN) leaders discussed measures to intensify cooperation and adopt a collective response to the pandemic. The leaders agreed on collaborative efforts to maintain trade and economic ties and support regional platforms and mechanisms. Furthermore, the leaders agreed to strengthen the region’s early warning system for pandemics, explore the establishment of a COVID-19 ASEAN response fund, and enhance timely data and information sharing, coordinate cross-border public health responses, and amplify cooperation around regional food security.^36^ ASEAN leaders agreed to escalate cooperation in sharing medical and health information and agreed on best practices to enhance emergency preparedness and response.

Regional cooperation plays an important role not only in sharing knowledge and best practices, but also in strengthening research and coordinating and developing critical treatment strategies, including effective interventions, vaccines, and other drugs.

In conclusion, COVID-19 is a truly global problem severely affecting every corner of the world. With overwhelmed health systems, countries must balance measures that address mortality resulting from COVID-19 with those addressing other diseases, including cancer. Asia Pacific is not immune to the impact of COVID-19 on public health and social systems. The overarching goal is to continue to provide safe, compassionate, and innovative care for patients with cancer. It is imperative that policymakers recognize people affected with cancer as a vulnerable group with special needs. Because of the profound economic integration of developing Asia with global trade and tourism, the region will weaken tremendously as a result of the socioeconomic effects of the pandemic. The ongoing crisis is an opportunity to strengthen regional cooperation. Joint action at subregional, regional, and global levels is imperative to share data, information, and experience and ensure the supply and delivery of medical materials and equipment. Building a stronger, more collaborative, and more resilient Asia Pacific region is crucial in meeting the challenges of the COVID-19 pandemic.

**TABLE 1:**
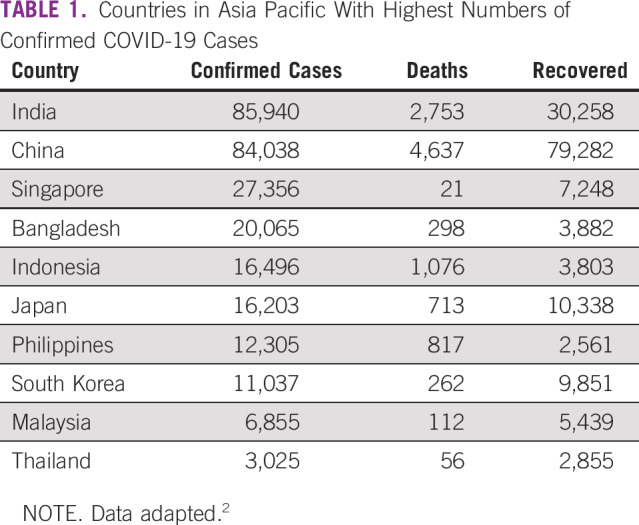
Countries in Asia Pacific With Highest Numbers of Confirmed COVID-19 Cases
